# Supplementary material for: Integrative analysis of genomic and transcriptomic characteristics associated with progression of aggressive thyroid cancer
Source: Nat Commun. 2019 Jun 24;10:2764. doi: 10.1038/s41467-019-10680-5 (PMC6591357; doi:10.1038/s41467-019-10680-5)
Supplement: Supplementary file 1 — Supplementary Information [file 41467_2019_10680_MOESM1_ESM.pdf]

**Supplementary Information**

# Integrative Analysis of Genomic and Transcriptomic Characteristics Associated with Progression of Aggressive Thyroid Cancer

Yoo *et al.*

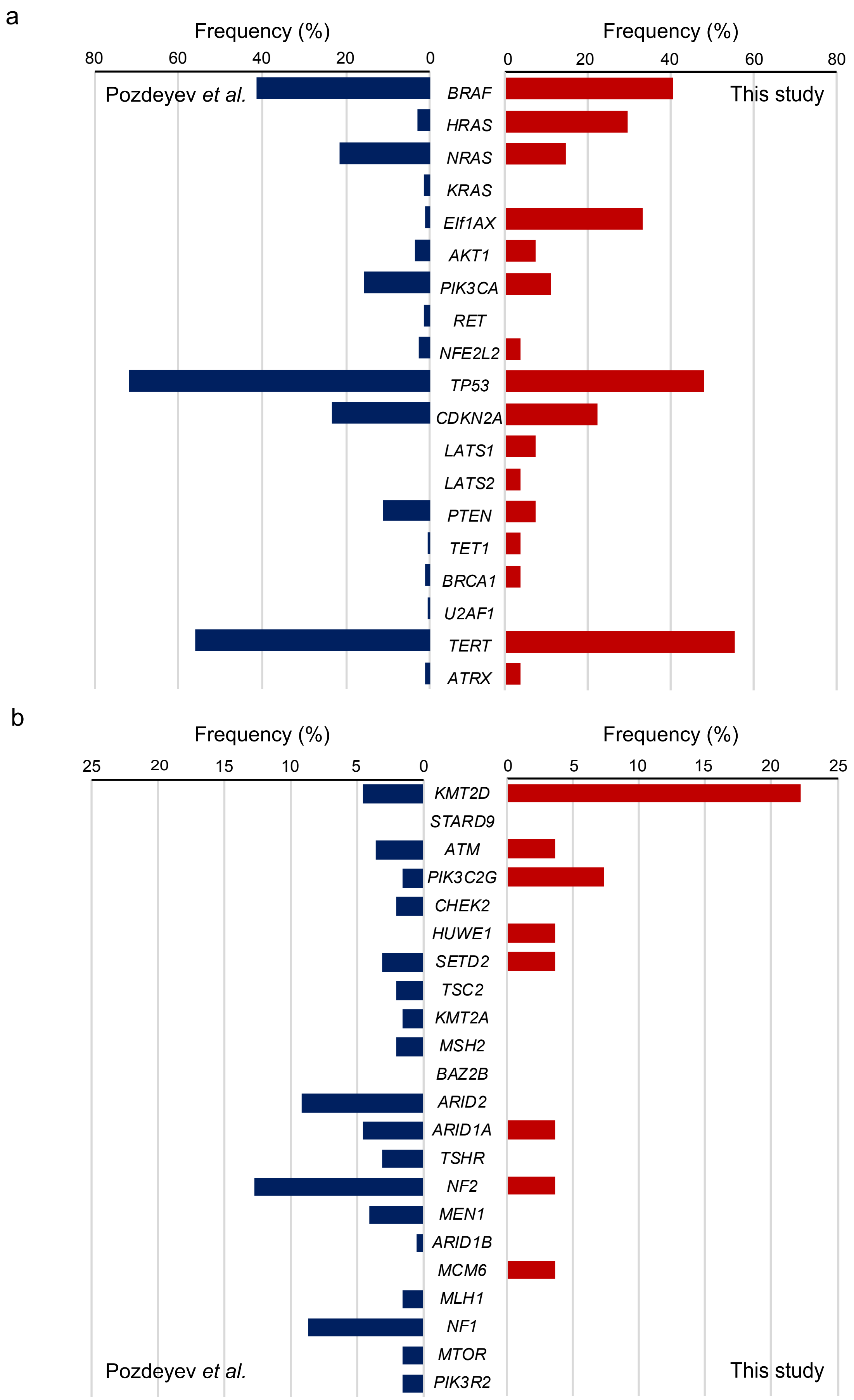


**Supplementary Figure 1. The comparison of gene alteration frequency of ATC.** Left and right bars represent the mutation frequencies from Pozdeyev *et al.* (left) and this study (right). a) Genes illustrated in Figure 1. b) Genes illustrated in Supplementary Figure 3.

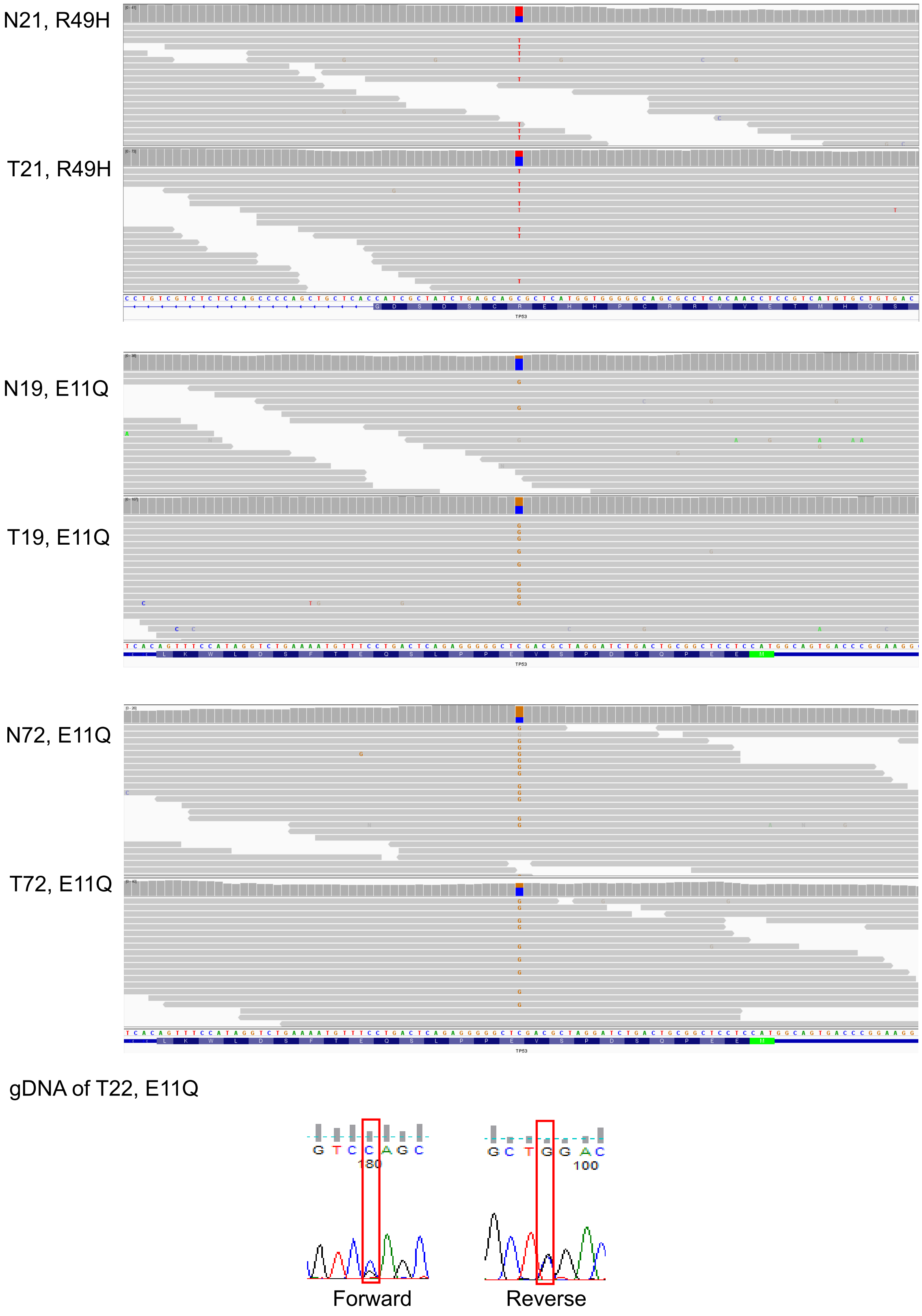


**Supplementary Figure 2. Germline *TP53* mutations in study subjects.**

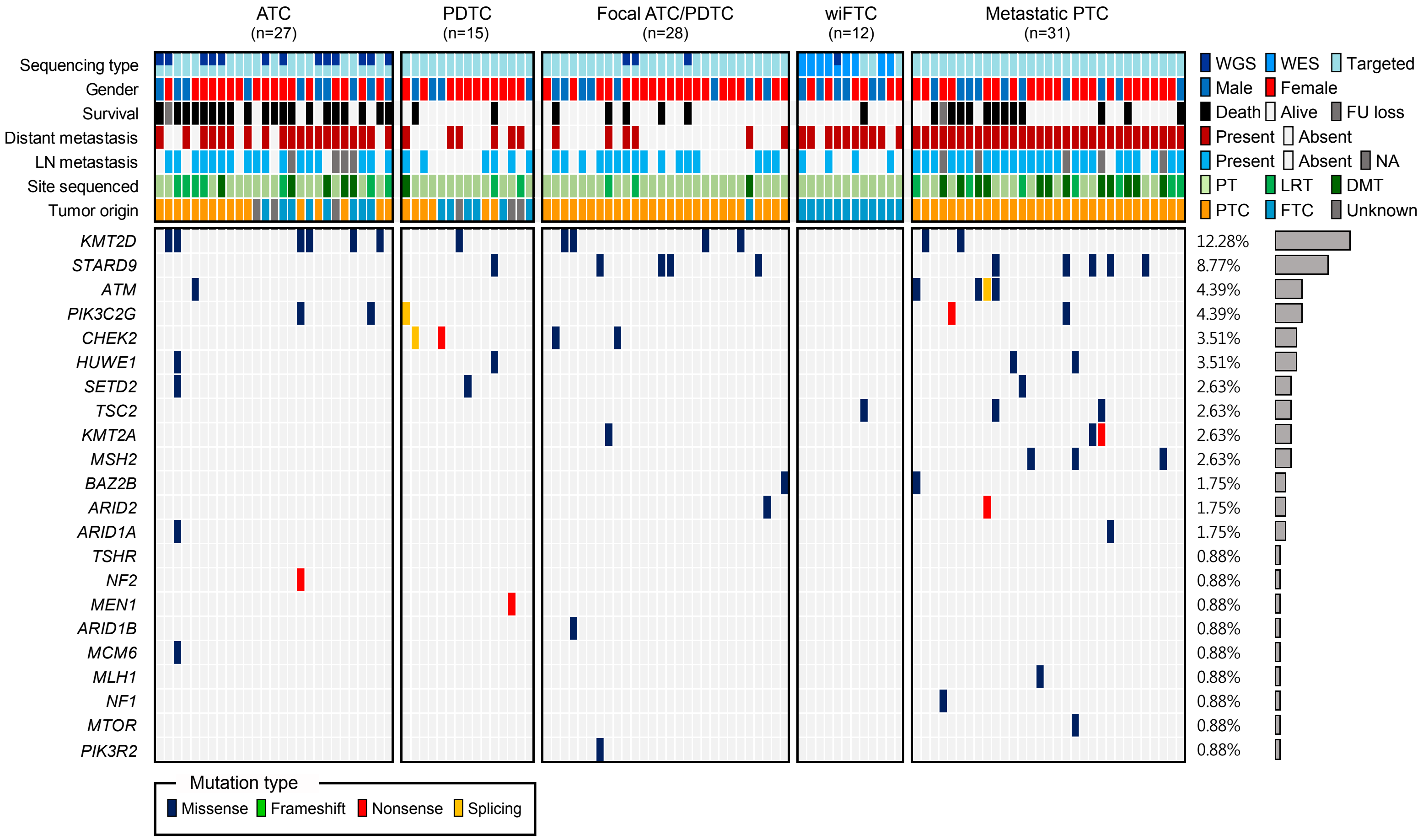


**Supplementary Figure 3. The putative genes associated with the progression of TC.** Samples were sorted as the same order of Figure 1.

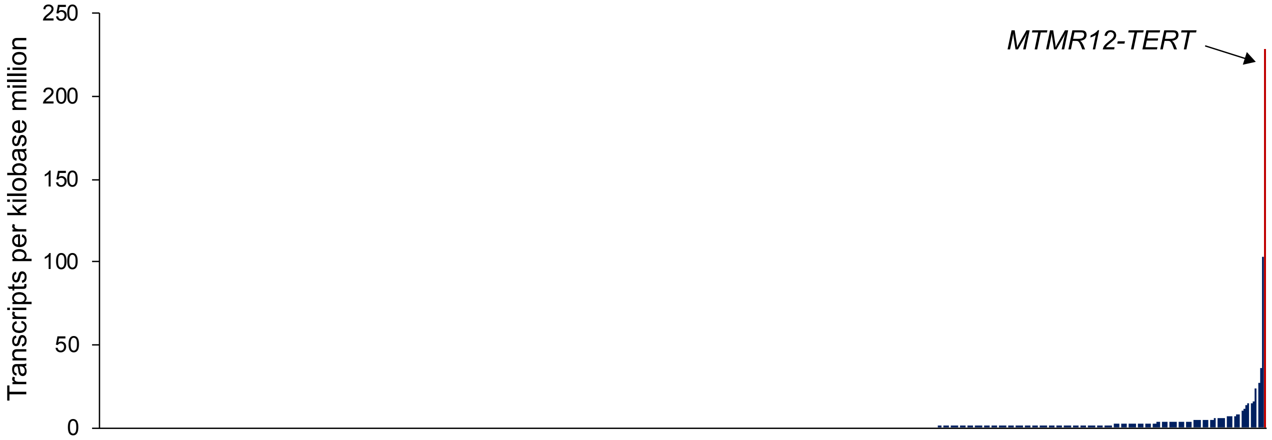


**Supplementary Figure 4. *TERT* gene expression level of TCGA samples.** Each bar represents *TERT* expression level of one sample. Samples were sorted by the order of *TERT* gene epxression level.

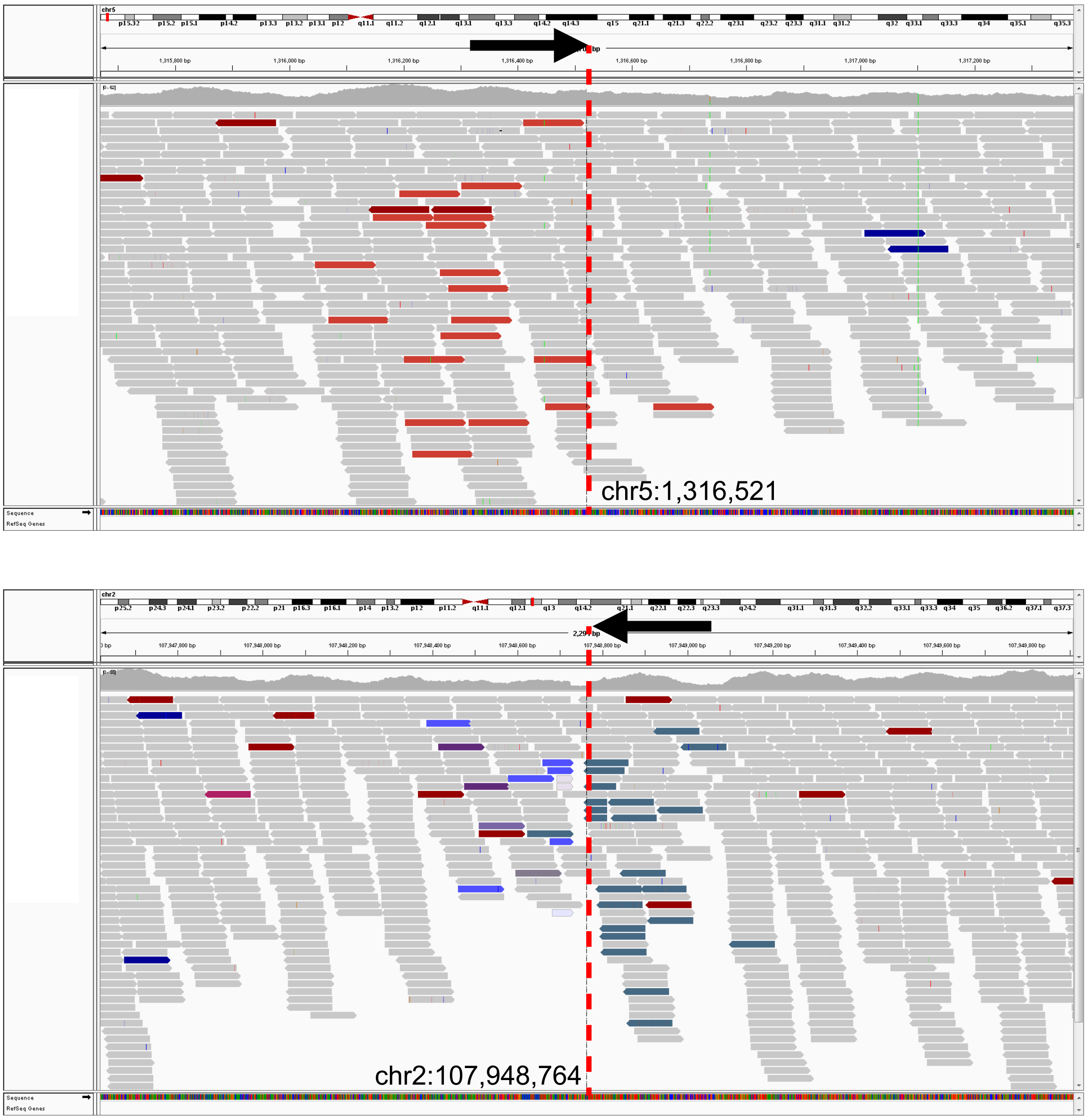


**Supplementary Figure 5. The inter-chromosomal translocation at *TERT* upstream region.** Upper and lower panels represent the whole-genome sequencing alignment at *TERT* upstream and partner regions, respectively.

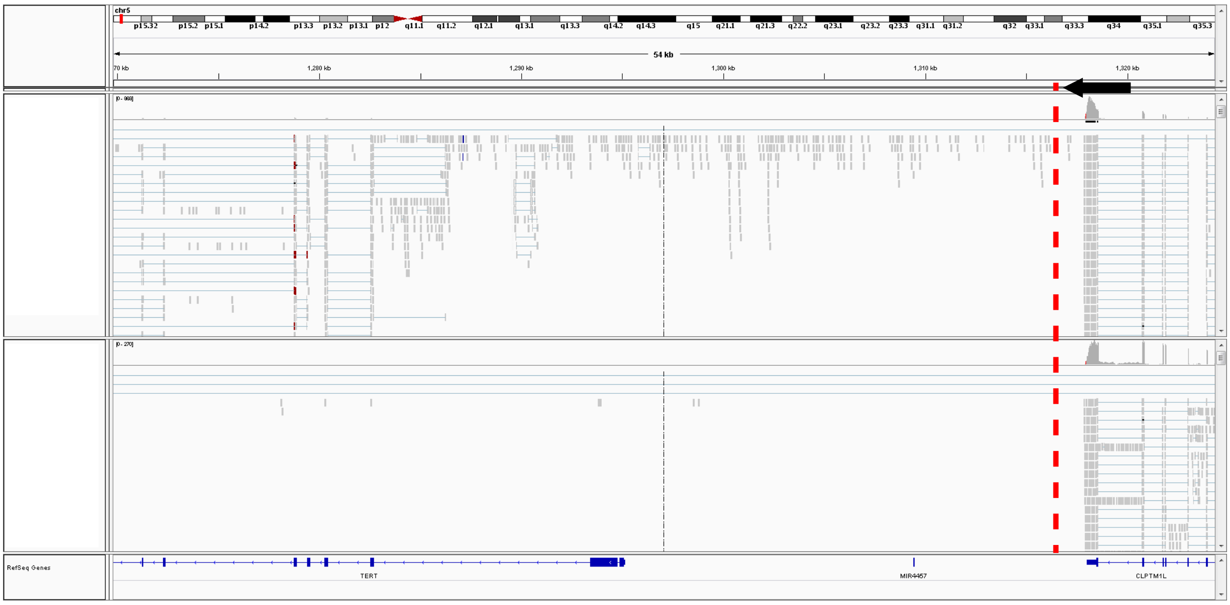


**Supplementary Figure 6. Abnormal RNA-seq alignment in intergenic region after translocation breakpoint at *TERT* upstream region.** Upper and lower panel represent data of tumor and normal thyroid tissue, respectively.

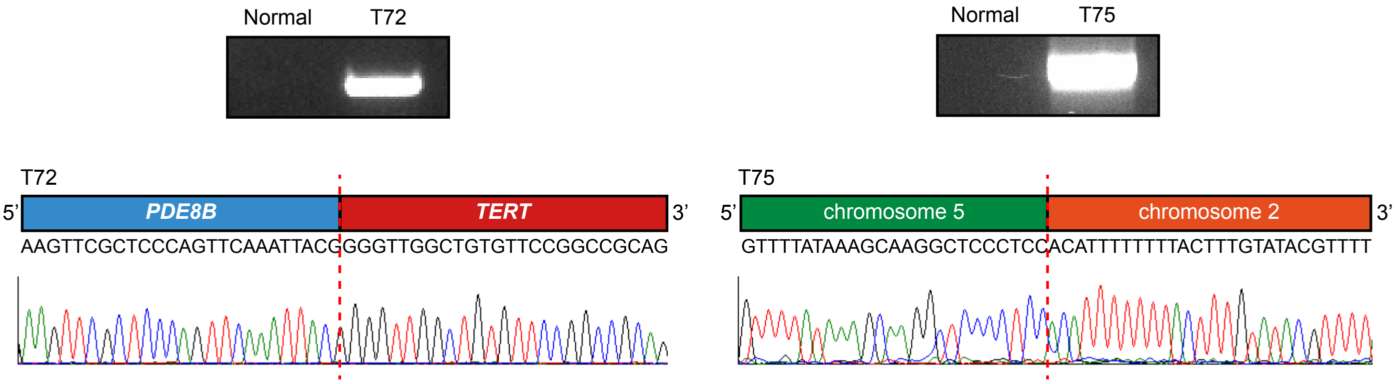


**Supplementary Figure 7. Validation of *TERT* rearrangements.** *PDE8B*-*TERT* fusion gene (left) and inter-chromosomal translocation of *TERT* upstream region (right).

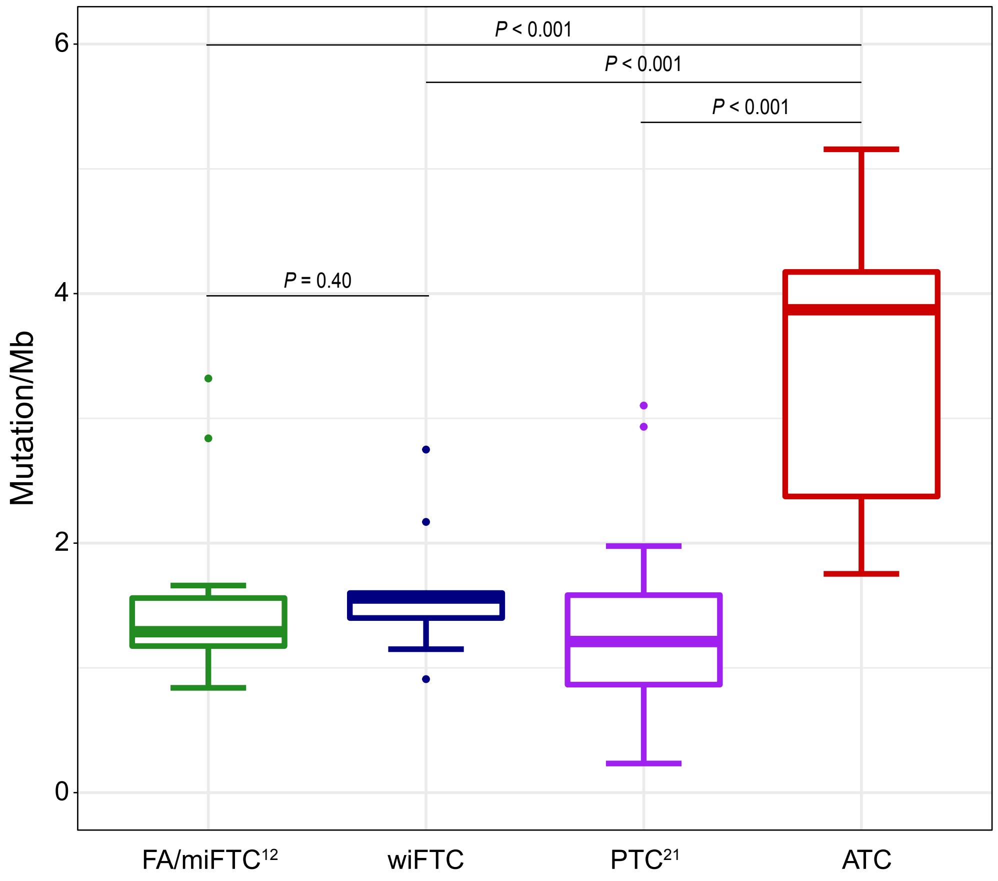


**Supplementary Figre 8. Tumor mutational burden in various types of TC.** Whole-exome sequenced FA/miFTC and PTC from Jung *et al.* and Rubinstein *et al.* were used to identify the somatic mutations in TCs. *P*-values from two-tailed Mann-Whitney U-test were represented.

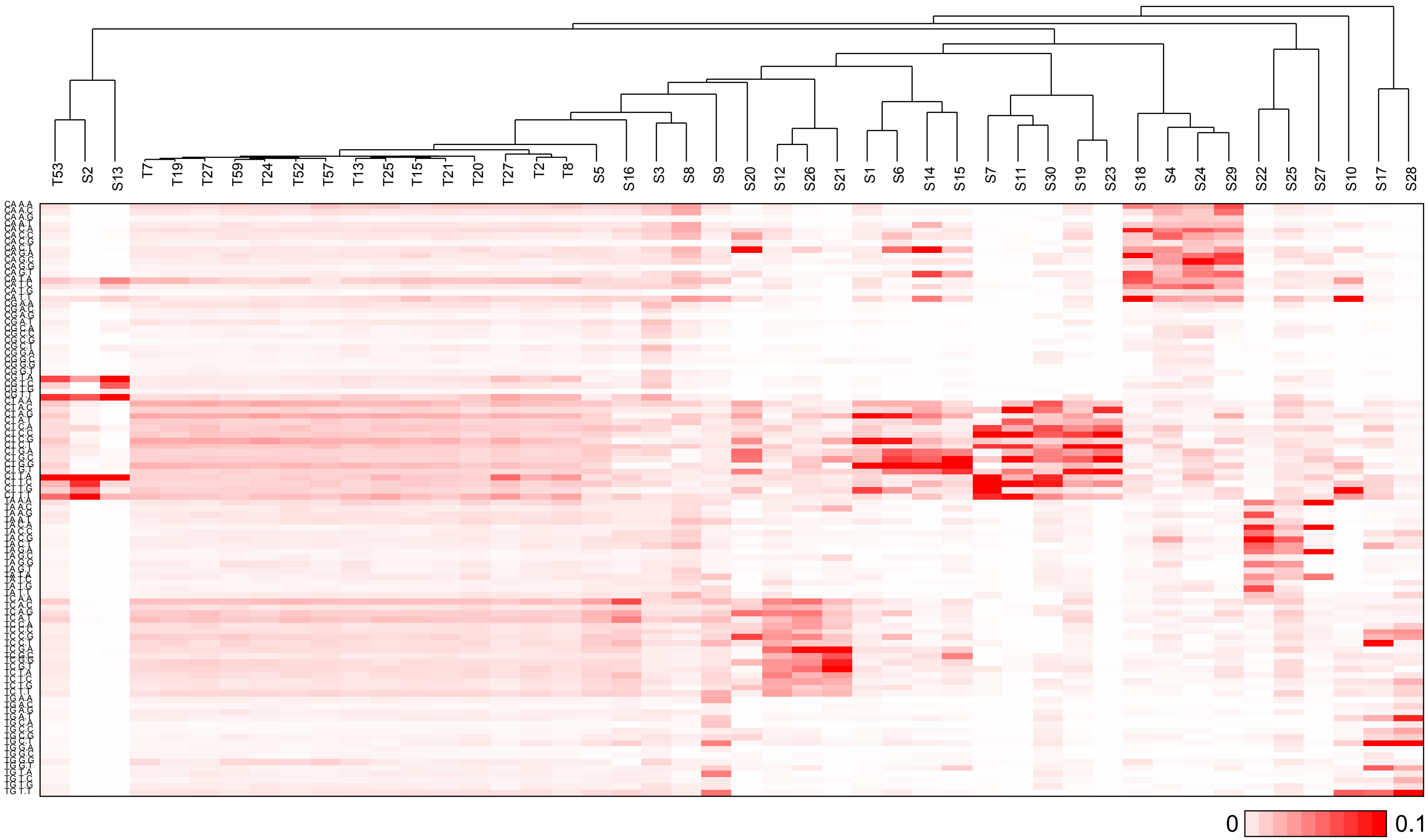


**Supplementary Figure 9. The mutational signature analysis.** The average-linkage hierarchical clustering was used. S denotes the reference signature from COSMIC.

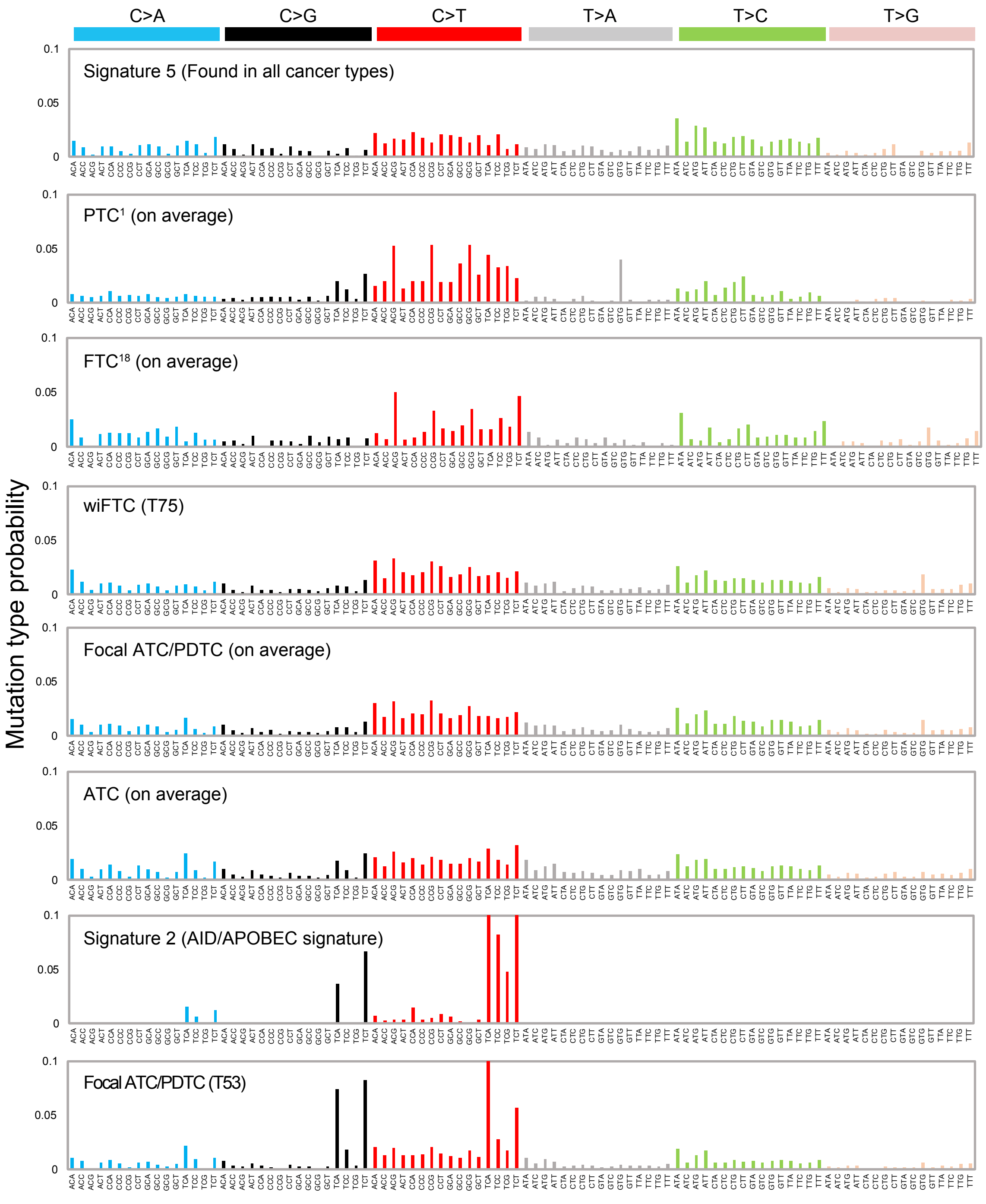


**Supplementary Figure 10. The representative mutational signatures in TC.** The average values of 96 motifs from whole-exome sequenced tumors in TCGA and Jung *et al.* were used for PTC and FTC. Signature 2 and 5 were downloaded from COSMIC.

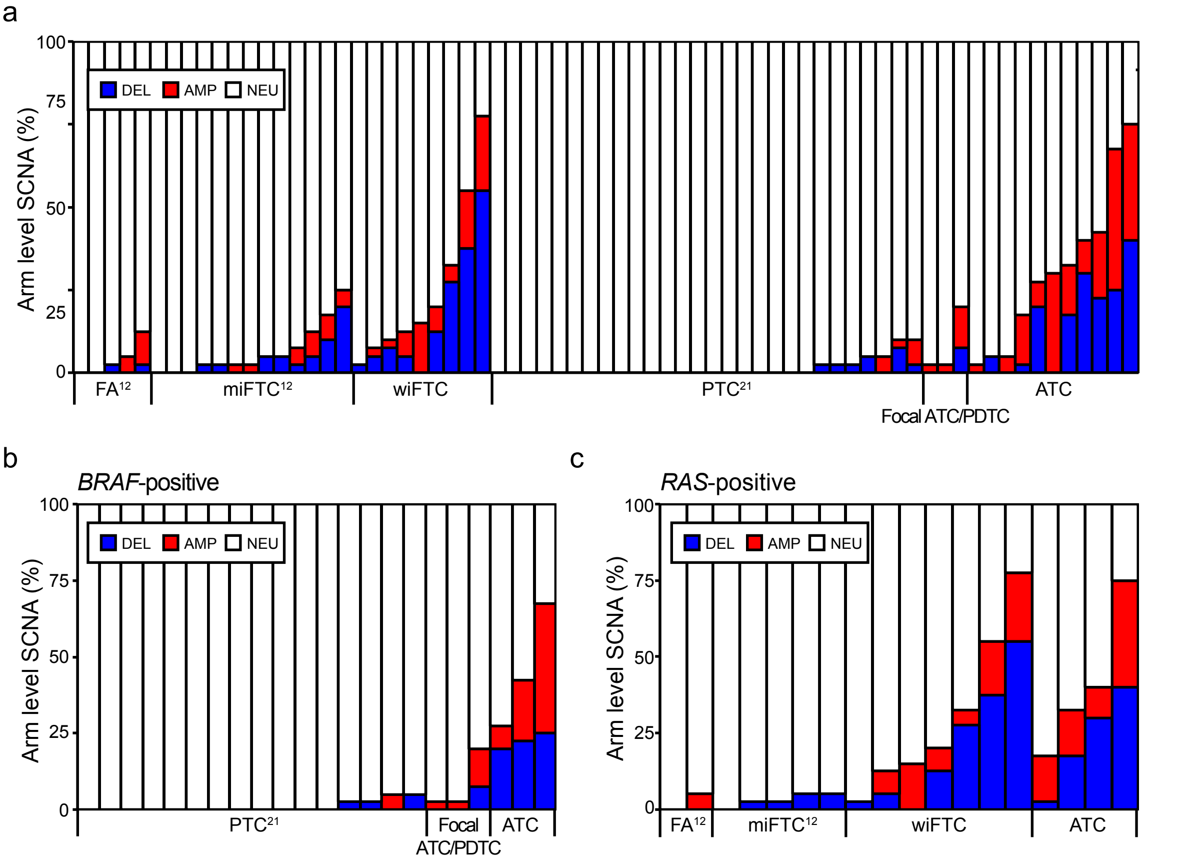


**Supplementary Figure 11. Arm-level SCNA in various types of TC.** a) Bar chart represents arm-level SCNA burden of TCs regardless of driver mutation. b) Arm-level SCNA in *BRAF*-positive TCs. c) Arm-level SCNA in *RAS*-positive TCs. Each bar represents individual tumor.

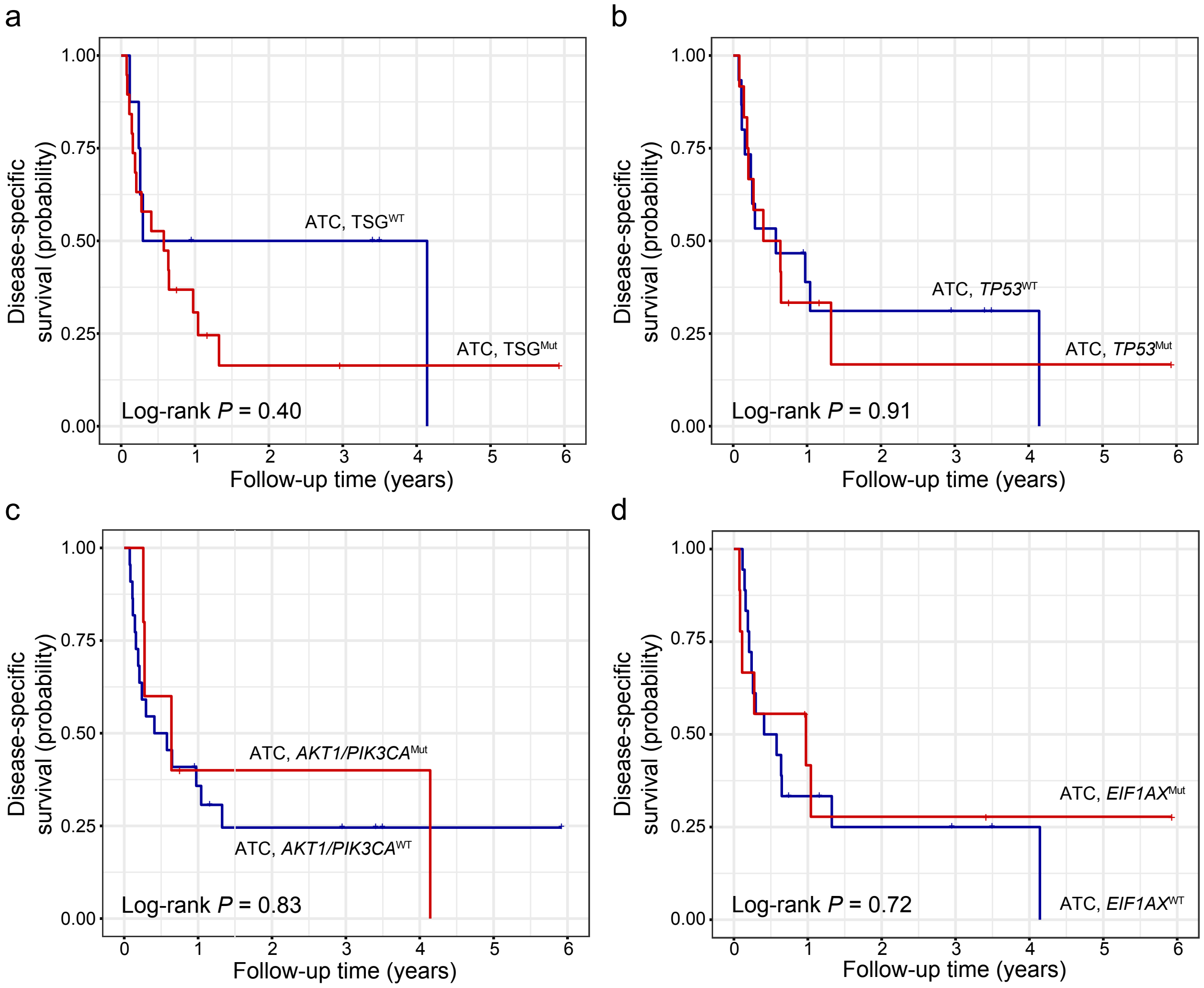


**Supplementary Figure 12. The effect of diverse types of mutation and disease-specific survival in ATC.**
a) TSG. b) *TP53*. c) *AKT1*/*PIK3CA* co-mutation. d) *EIF1AX* co-mutation.

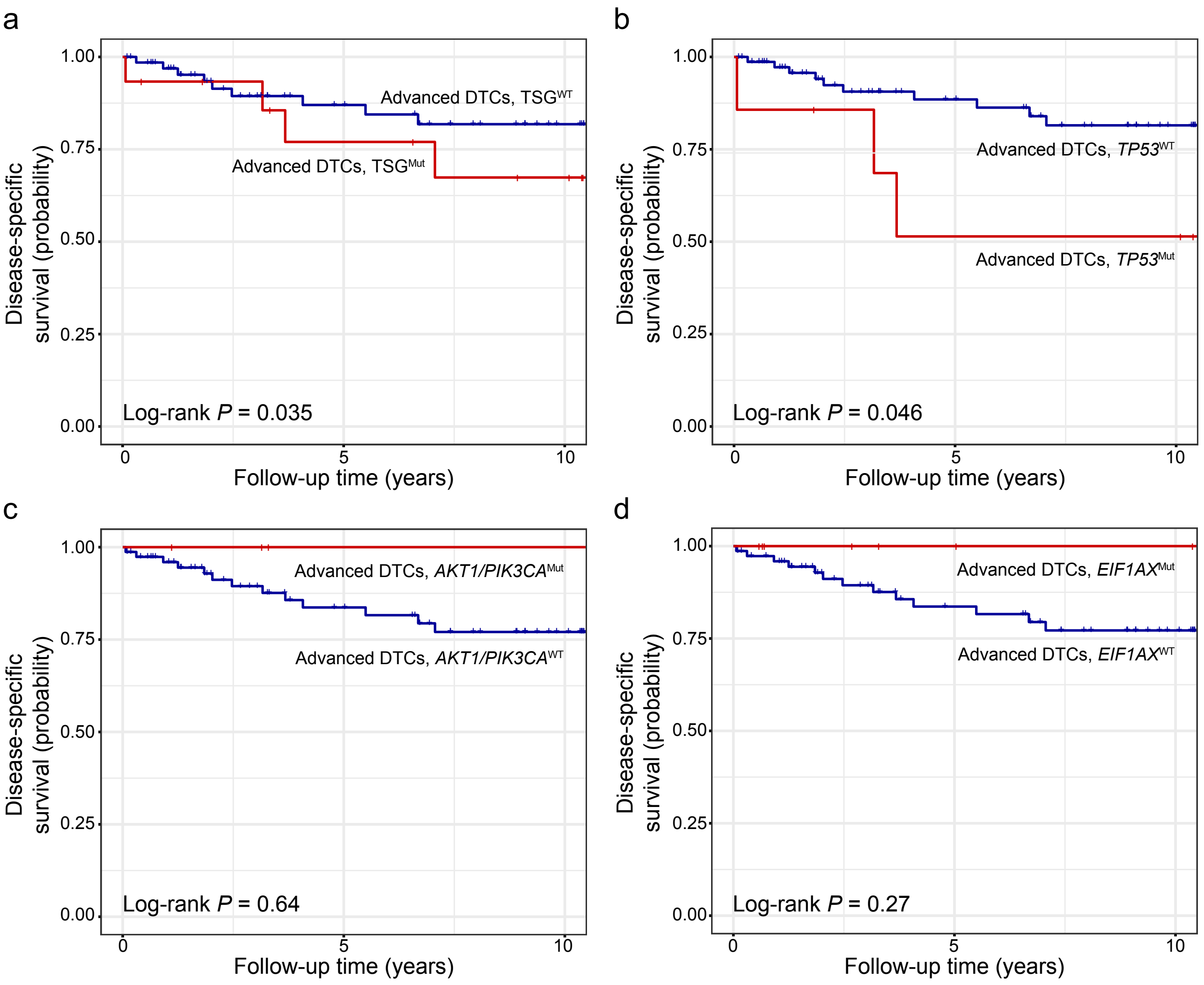


**Supplementary Figure 13. The effect of diverse types of mutation and disease-specific survival in advanced TCs.**
a) TSG. b) *TP53*. c) *AKT1/PIK3CA* co-mutation. d) *EIF1AX* co-mutation.

a

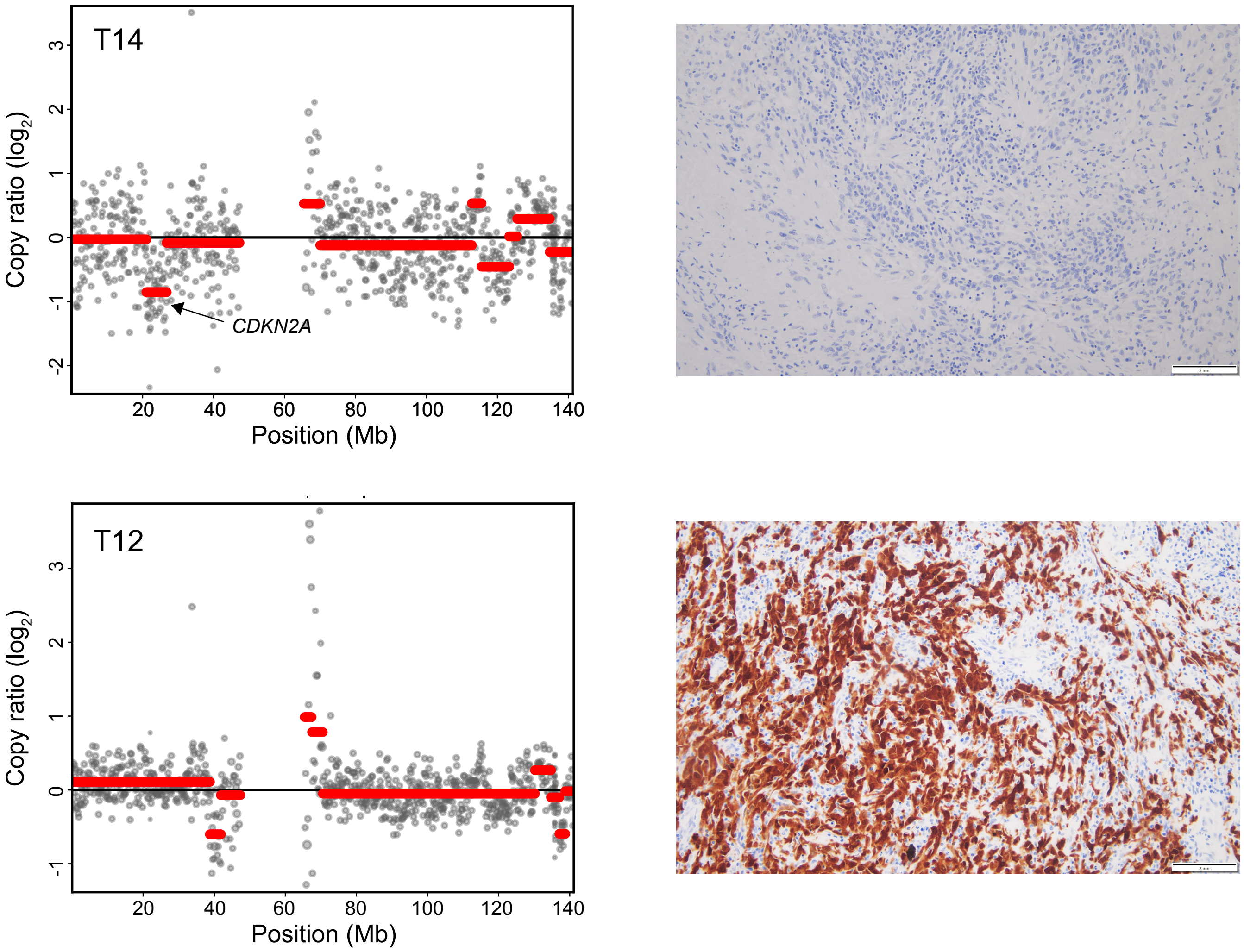


b

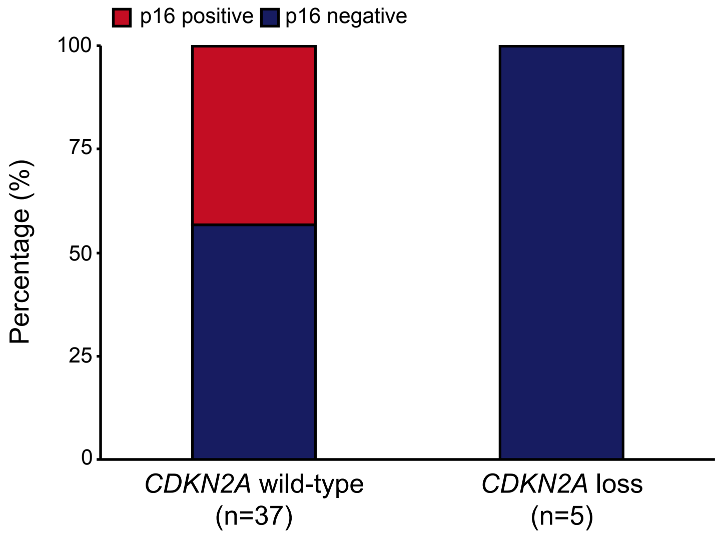


**Supplementary Figure 14. p16 immunohistochemistry using tissue microarray analysis.** a) The representative images of p16-negative (upper) and p16-positive (lower) results. b) The relationship between *CDKN2A* loss and p16 expression.

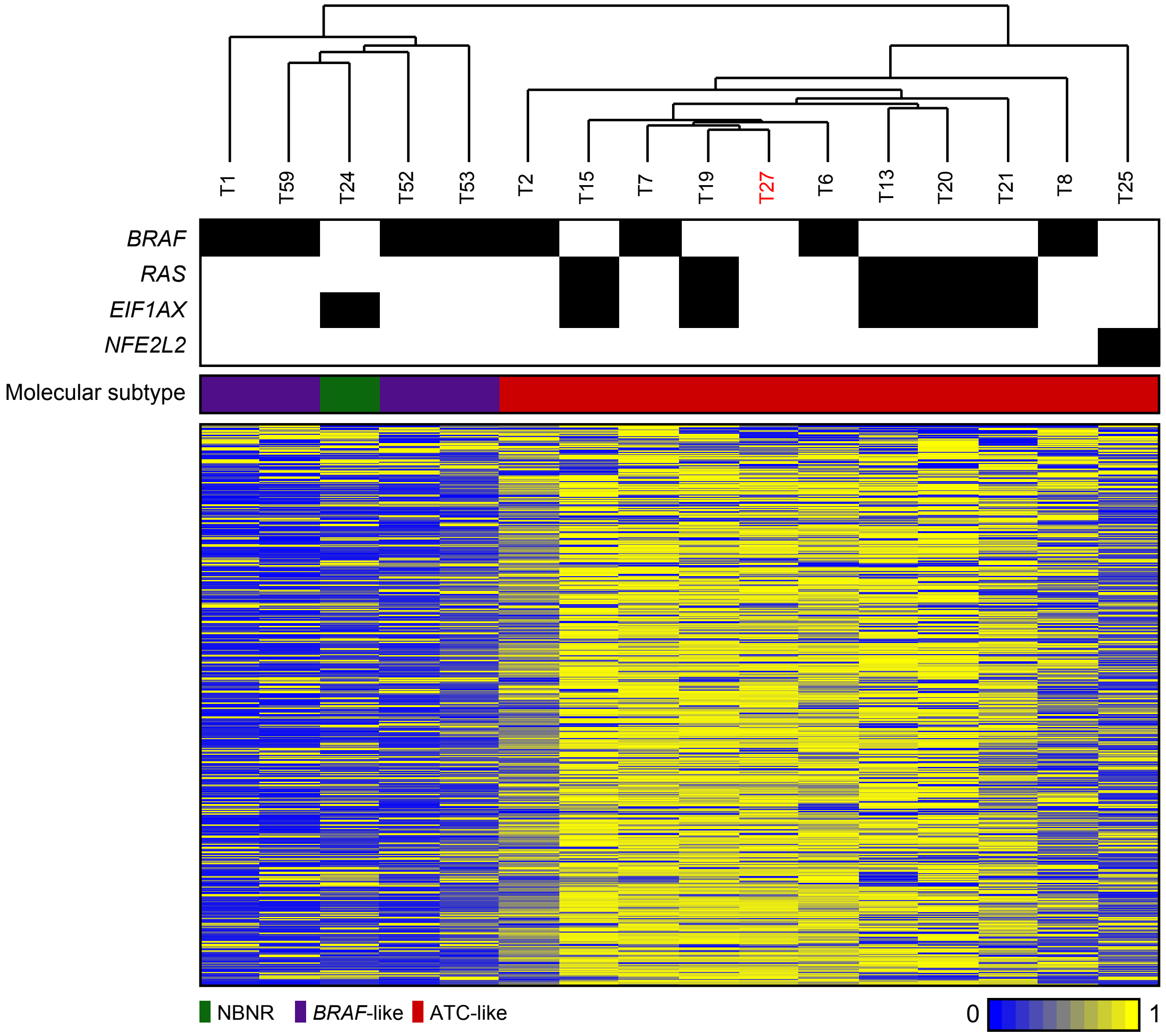


**Supplementary Figure 15. DNA methylation profile of ATC.** The 500 most variable CpG sites were displayed by heatmap.Samples were clustered by the average-linkage hierarchical clustering method. T27 which do not have any mutation in cancer related gene was colored by red.

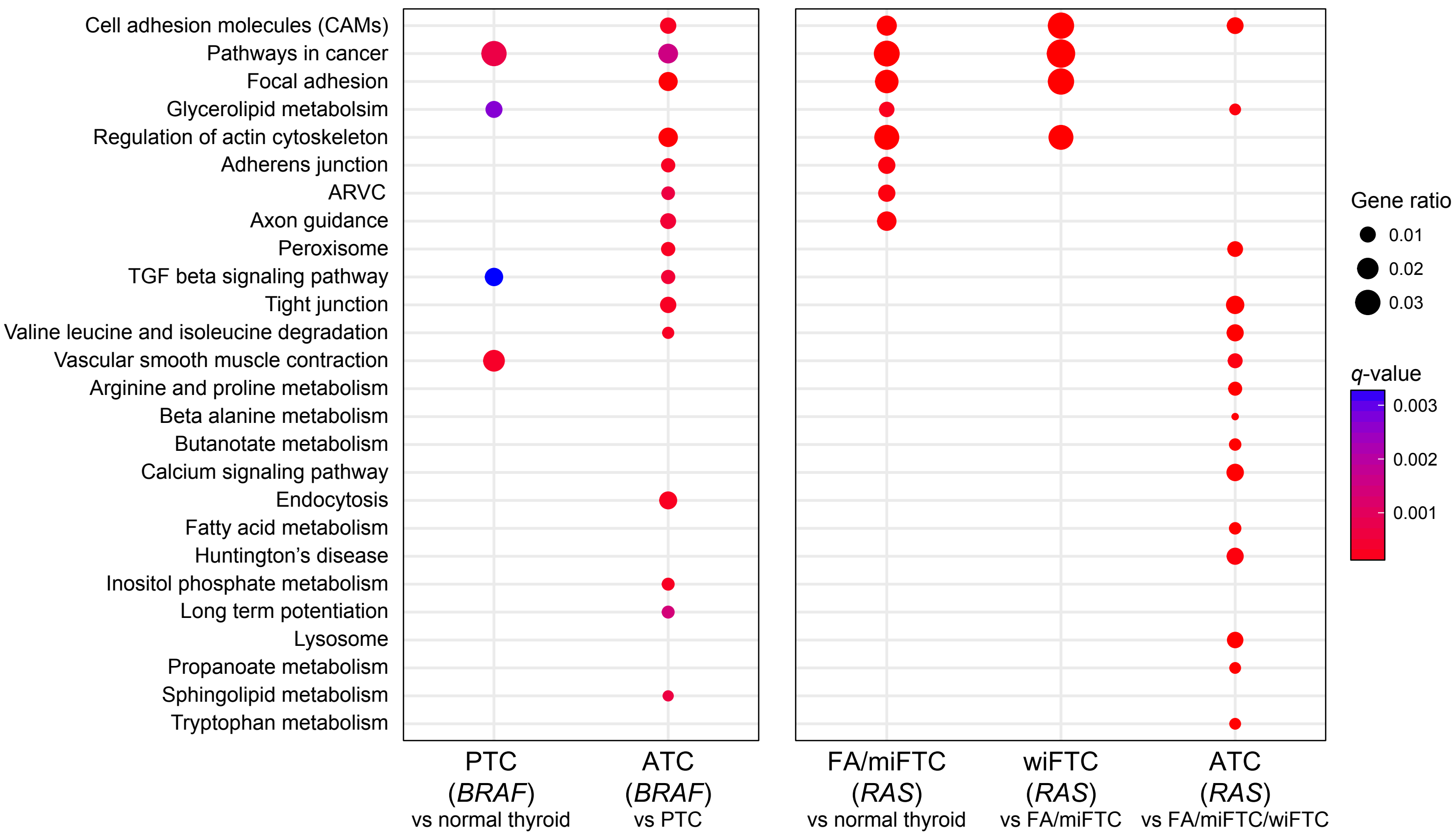


**Supplementary Figure 16. The top 15 signiicantly down-regulated KEGG pathways in *BRAF*- and *RAS*-positive ATCs.** The significance of these pathways were also marked in PTC, FA/miFTC, and wiFTC, when they were also found within the top 15 significantly down-regulated pathways of each tumor.

**Supplementary Table 1. The regions with significant somatic copy number alteration in ATC.**

| Type of region | Cytoband | Wide peak boundaries | Genes in wide peak | Residual $q$-value[a] |
|---|---|---|---|---|
| Amplification | 2q21.2 | chr2:133,034,593-133,118,006 | *ANKRD30BL* | 0.0001 |
| | 16q22.2 | chr16:70,882,524-71,200,029 | *HYDIN* | 0.0001 |
| | 6p11.2 | chr6:57,212,739-57,568,050 | *hsa-mir-548u, PRIM2* | 0.0001 |
| | 15q26.3 | chr15:102,504,813-102,531,392 | *WASH3P, DDX11L1, DDX11L9* | 0.0001 |
| | 1p36.13 | chr1:16,837,477-16,838,671 | *CROCCP3* | 0.0007 |
| | 1q21.1 | chr1:145,374,344-145,380,272 | *NBPF10* | 0.0011 |
| | 7q11.22 | chr7:71,563,279-71,589,549 | *CALN1* | 0.0047 |
| | 17q12 | chr17:36,269,518-36,405,553 | *TBC1D3F, LOC440434, TBC1D3* | 0.0047 |
| | 2p11.1 | chr2:91,676,990-91,823,234 | *LOC654342* | 0.0051 |
| | 14q11.2 | chr14:19,385,975-20,162,672 | *POTEG, POTEM, LOC642426* | 0.0093 |
| | 10q11.22 | chr10:47,021,072-47,104,171 | *PPYR1, LOC643650* | 0.0216 |
| Deletion | 9p21.3 | chr9:22,002,865-22,009,400 | *CDKN2A, CDKN2B* | 5.88E-07 |
| | 2q36.1 | chr2:223,808,117-224,461,675 | *KCNE4* | 0.0004 |
| | 19p13.3 | chr19:1,279,242-1,355,035 | *ENFA2* | 0.0194 |
| | 8p23.1 | chr8:7,439,997-7,946,469 | *DEFB4A, SPAG11B, DEFB103B, DEFB104A, DEFB105A, DEFB106A, DEFB107A, DEFB103A, FAM90A13, FAM90A8, FAM90A18, FAM90A9, FAM90A10, DEFB107B, DEFB104B, DEFB106B, DEFB105B, DEFB109P1B, FAM90A14, SPAG11A, FAM90A19, FAM66E, LOC100132396* | 0.0194 |
| | 22q13.32 | chr22:48,934,669-48,943,223 | *LOC284933* | 0.0194 |
| | 9q22.2 | chr9:92,221,450-92,782,978 | *UNQ6494* | 0.0283 |
| | 17p11.2 | chr17:21,826,480-22,022,469 | *FLJ36000* | 0.0213 |
| | 21q22.3 | chr21:46,046,559-46,048,316 | *KRTAP10-9* | 0.0371 |

[a] The residual $q$-value represents the significance of peak region after excluding overlapped amplifications and deletions in other, more significant peak regions.

**Supplementary Table 2. Hazard ratios (HR) of *CDKN2A* loss for death in ATC and advanced DTCs.**

| Model | All (n=113) | | ATC (n=27) | | Advanced DTCs (n=86) | |
|---|---|---|---|---|---|---|
| | HR (95% CI) | *P* | HR (95% CI) | *P* | HR (95% CI) | *P* |
| Model 1 | 11. 03 (4.97-24.45) | <0.001 | 2.95 (1.08-8.04) | 0.034 | 31.36 (7.71-127.60) | <0.001 |
| Model 2 | 13.59 (5.54-33.37) | <0.001 | 4.47 (1.33-15.01) | 0.016 | 21.48 (4.56-101.14) | <0.001 |
| Model 3 | 10.56 (4.29-25.96) | <0.001 | 3.90 (1.10-13.78) | 0.035 | 15.00 (3.15-71.46) | <0.001 |
| Model 4 | 9.61 (3.73-24.79) | <0.001 | 6.67 (1.34-33.12) | 0.02 | 9.88 (1.97-49.57) | <0.001 |

Model 1. Unadjusted.

Model 2. Adjusted for age at surgery for analyzed tissue and sex.

Model 3. Adjusted for age at surgery for analyzed tissue, sex, and distant metastasis.

Model 4. Adjusted for age at surgery for analyzed tissue, sex, distant metastasis, and tumor origin.

**Supplementary Table 3. Hazard ratios (HR) of p16 expression for death in ATC.**

| Model | All (n=57) | | ATC (n=17) | |
|---|---|---|---|---|
| | HR (95% CI) | *P* | HR (95% CI) | *P* |
| Model 1 | 13.86 (1.83-105.20) | 0.011 | 7.10 (0.890-56.04) | 0.063 |
| Model 2 | 10.67 (0.09-1.38) | 0.023 | 35.25 (1.38-898.79) | 0.031 |
| Model 3 | 2.58 (0.25-26.22) | 0.424 | 30.05 (0.93-973.91) | 0.056 |
| Model 4 | 1.13 (0.31-30.72) | 0.333 | 5.17 (0.29-93.11) | 0.266 |

Model 1. Unadjusted.
Model 2. Adjusted for age at surgery for analyzed tissue and sex.
Model 3. Adjusted for age at surgery for analyzed tissue, sex, and distant metastasis.
Model 4. Adjusted for age at surgery for analyzed tissue, sex, distant metastasis, and tumor origin.

**Supplementary Table 4. The list of genes captured by custom probes.**

| Target | Gene symbols |
|---|---|
| Small size mutations | *AKT1, AKT3, ARID1A, ARID1B, ARID2, ARID5B, ATM, ATRX, BAZ2B*[a] *, BRAF, CDKN2A, CHEK2, CTNNA2, DICER1, EIF1AX, EZH1, HRAS, HUWE1*[a] *, IDH1, KMT2A, KMT2C, KMT2D, KRAS, LATS1, LATS2, MCM6*[a] *, MEN1, MLH1, MSH2, MSH6, MTOR, NF1, NF2, NFE2L2, NRAS, PBRM1, PIK3C2G, PIK3C3, PIK3CA, PIK3CG, PIK3R1, PIK3R2, PPM1D, PTEN, RB1, SETD2, SMARCB1, SOS1, SPOP, STARD9*[a] *, STK11, TET1, TP53, TSC1, TSC2, TSHR, U2AF1* |
| Fusion genes | *ALK, B4GALNT3, BRD4, FGFR2, FGFR3, MET, NTRK1, NTRK3, NUTM1, PAX8, RET, THADA* |
| Promoter mutations | *TERT* |

[a]Genes from our unpublished work about distant metastasis of FTC.

**Supplementary Table 5. Nucleotide sequences of primers used for PCR.**

| Target | Primers | Nucleotide sequence |
|---|---|---|
| *TERT* promoter (Primter #1) | Forward | CACCCGTCCTGCCCCTTCACCTT |
| | Reverse | CTTCCCACGTGCGCAGCAGGA |
| *TERT* promoter (Primer #2) | Forward | CCCTTCACCTTCCAGCTC |
| | Reverse | CAGCGCTGCCTGAAACTC |
| *TERT* upstream translocation | Forward | ACTCCTTTCCCGTTTGTGTG |
| | Reverse | GAAGACAGGTGGCAGAGAGG |
| *PDE8B-TERT* | Forward | ATCGGATGACCATGAAGAGG |
| | Reverse | ACACTCATCAGCCAGTGCAG |
| *TP53 (*E11Q ) | Forward | CAGCCATTCTTTTCCTGCTC |
| | Reverse | TCCCACAGGTCTCTGCTAGG |
| *TP53* (R49H) | Forward | GTTTCTTTGCTGCCGTCTTC |
| | Reverse | ACACGCAAATTTCCTTCCAC |
| *BCL2L1* | Forward | CCTCTCCCGACCTGTGATAC |
| | Reverse | CCAAAACACCTGCTCACTCA |
| *SOCS3* | Forward | AGGCTCCTTTGTGGACTTCA |
| | Reverse | AACTTGCTGTGGGTGACCAT |
| *MYC* | Forward | GAGGCTATTCTGCCCATTTG |
| | Reverse | CACCGAGTCGTAGTCGAGGT |
| β-ACTIN | Forward | TGACGTGGACATCCGCAAAG |
| | Reverse | CTGGAAGGTGGACAGCGAGG |
