# Supplementary material for: Integrative analysis of genomic and transcriptomic characteristics associated with progression of aggressive thyroid cancer
Source: Nat Commun. 2019 Jun 24;10:2764. doi: 10.1038/s41467-019-10680-5 (PMC6591357; doi:10.1038/s41467-019-10680-5)
Supplement: Supplementary file 3 — Description of Additional Supplementary Files [file 41467_2019_10680_MOESM3_ESM.pdf]

Description of Additional Supplementary Files

File Name: Supplementary Data 1
Description: Variant detection concordance between WGS and targeted sequencing

File Name: Supplementary Data 2
Description: Patient information
