# Supplementary material for: Integrative analysis of genomic and transcriptomic characteristics associated with progression of aggressive thyroid cancer
Source: Nat Commun. 2019 Jun 24;10:2764. doi: 10.1038/s41467-019-10680-5 (PMC6591357; doi:10.1038/s41467-019-10680-5)
Supplement: Supplementary file 6 — Reporting Summary [file 41467_2019_10680_MOESM6_ESM.pdf]

nature research

Corresponding author(s): Jeong-Sun Seo
Young Joo Park

Last updated by author(s): Feb 28, 2019

Reporting Summary

Nature Research wishes to improve the reproducibility of the work that we publish. This form provides structure for consistency and transparency in reporting. For further information on Nature Research policies, see Authors & Referees and the Editorial Policy Checklist.

Statistics

For all statistical analyses, confirm that the following items are present in the figure legend, table legend, main text, or Methods section.

n/a	Confirmed	
☐	☒	The exact sample size (*n*) for each experimental group/condition, given as a discrete number and unit of measurement
☐	☒	A statement on whether measurements were taken from distinct samples or whether the same sample was measured repeatedly
☐	☒	The statistical test(s) used AND whether they are one- or two-sided<br>*Only common tests should be described solely by name; describe more complex techniques in the Methods section.*
☐	☒	A description of all covariates tested
☐	☒	A description of any assumptions or corrections, such as tests of normality and adjustment for multiple comparisons
☐	☒	A full description of the statistical parameters including central tendency (e.g. means) or other basic estimates (e.g. regression coefficient) AND variation (e.g. standard deviation) or associated estimates of uncertainty (e.g. confidence intervals)
☐	☒	For null hypothesis testing, the test statistic (e.g. *F*, *t*, *r*) with confidence intervals, effect sizes, degrees of freedom and *P* value noted<br>*Give P values as exact values whenever suitable.*
☒	☐	For Bayesian analysis, information on the choice of priors and Markov chain Monte Carlo settings
☒	☐	For hierarchical and complex designs, identification of the appropriate level for tests and full reporting of outcomes
☒	☐	Estimates of effect sizes (e.g. Cohen's *d*, Pearson's *r*), indicating how they were calculated

Our web collection on statistics for biologists contains articles on many of the points above.

Software and code

Policy information about availability of computer code

Data collection	All the softwares used in this study were described in Methods section.
Data analysis	BWA (0.7.15), STAR (2.4.1c), GATK (3.8.0), Picard tools (1.129), MuTect (1.1.7), GISTIC2 (2.0.23), FACETS (0.5.0), R (3.3), EXCAVATOR2 (1.1.2), CNVKit (0.9.3.dev0), HTSeq (0.6.1p1), DESeq2 (1.14.1), MOJO (0.0.5), bowtie (0.1.1), MACS (1.4.2), ROSE, SomaticSignatures (2.1.0), GenePattern (3.8.0)

For manuscripts utilizing custom algorithms or software that are central to the research but not yet described in published literature, software must be made available to editors/reviewers. We strongly encourage code deposition in a community repository (e.g. GitHub). See the Nature Research guidelines for submitting code & software for further information.

Data

Policy information about availability of data

All manuscripts must include a data availability statement. This statement should provide the following information, where applicable:
- Accession codes, unique identifiers, or web links for publicly available datasets
- A list of figures that have associated raw data
- A description of any restrictions on data availability

All sequencing reads were submitted the European Genome Phenome Archive (https://www.ebi.ac.uk/ega/) with accession number EGAS00001003540.

Field-specific reporting

Please select the one below that is the best fit for your research. If you are not sure, read the appropriate sections before making your selection.

☒ Life sciences ☐ Behavioural & social sciences ☐ Ecological, evolutionary & environmental sciences

For a reference copy of the document with all sections, see nature.com/documents/nr-reporting-summary-flat.pdf

Life sciences study design

All studies must disclose on these points even when the disclosure is negative.

Sample size	Sample sizes were not calculated or included for this study.
Data exclusions	None.
Replication	Cell line experiments were performed with at least four replicates.
Randomization	Not relevant to the present study.
Blinding	Not relevant to the present study.

Reporting for specific materials, systems and methods

We require information from authors about some types of materials, experimental systems and methods used in many studies. Here, indicate whether each material, system or method listed is relevant to your study. If you are not sure if a list item applies to your research, read the appropriate section before selecting a response.

Materials & experimental systems

n/a	Involved in the study	
☒	☐	Antibodies
☐	☒	Eukaryotic cell lines
☒	☐	Palaeontology
☒	☐	Animals and other organisms
☐	☒	Human research participants
☒	☐	Clinical data

Methods

n/a	Involved in the study	
☒	☐	ChIP-seq
☒	☐	Flow cytometry
☒	☐	MRI-based neuroimaging

Eukaryotic cell lines

Policy information about cell lines

Cell line source(s)	CAL62, a human ATC cell line were provided by Dr. Yong-Hyun Jeon (Kyungpook National University Hospital, Daegu, Republic of Korea).
Authentication	Cell line was authenticated with STR analysis.
Mycoplasma contamination	Cell line was not tested for mycoplasma contamination, but there was no signs of it.
Commonly misidentified lines (See ICLAC register)	Not applicable.

Human research participants

Policy information about studies involving human research participants

Population characteristics	113 Korean patients with advanced thyroid cancer were included.
Recruitment	The patients were recruited at Seoul National University Hospital.
Ethics oversight	This study was approved by the institutional review board of Seoul National University Hospital, in accordance with the Declaration of Helsinki (approved ID: H-1307-034-501).

Note that full information on the approval of the study protocol must also be provided in the manuscript.
